# The p48MW Flow Diverter—Initial Human Experience

**DOI:** 10.1007/s00062-019-00827-8

**Published:** 2019-08-21

**Authors:** P. Bhogal, C. Bleise, J. Chudyk, I. Lylyk, R. Viso, N. Perez, H. Henkes, P. Lylyk

**Affiliations:** 1 The Royal London Hospital, Whitechapel Road, E1 1BB London, UK; 2 Neuroradiological Clinic, Clinica Sagrada Familia, ENERI, Buenos Aires, Argentina; 3 Medical Faculty, University Duisburg-Essen, Essen, Germany; 4 Neuroradiological Clinic, Klinikum Stuttgart, Stuttgart, Germany

**Keywords:** Stent, Flow diverter, P48 MW, Aneurysm, New devices

## Abstract

**Background and Purpose:**

The use of flow diverters to treat aneurysms arising from small caliber parent vessels has been reported. This article reports the results of the first in experiences with the p48MW (p48 Movable Wire) in humans, a device specifically designed to target vessels 1.75–3 mm in diameter.

**Methods:**

This monocentric study retrospectively reviewed the prospectively maintained database to identify all patients treated with the p48MW device between January 2017 and January 2019 at this institution. Patient demographics, aneurysm characteristics, angiographic and clinical follow-up were recorded as well as complications.

**Results:**

A total of 25 patients (20 female) with an average age of 55 ± 12.9 years (range 34–84) with 25 aneurysms were identified. The majority of the aneurysms was located in the anterior circulation (19/25, 76%). The average aneurysm dome width was 3.98 ± 3.6 mm (range 1.2–13 mm). Complete occlusion was seen in 18/24 (75%) aneurysms with neck remnants in 1/24 (4.2%) and continued aneurysm filling seen in the remaining cases (5/24, 20.8%). Adequate occlusion was seen in 79.2% of aneurysms (Raymond Roy Classification [RRC] grade I or II) during the follow-up period. There was a single technical complication with inappropriate deployment of the first p48MW. There was a single clinical complication (4%); however, the patient made a complete recovery (modified Rankin Scale [mRS] 0) and one patient died secondary to uncontrollable status epilepticus following acute subarachnoid hemorrhage unrelated to the treatment.

**Conclusion:**

The p48MW is safe and effective for the treatment of aneurysms including those arising from distal vessels.

## Introduction

The introduction of flow-diverting technology represented a paradigm shift in the way intracranial aneurysms could be treated. These new stents were originally tested in previously untreatable aneurysms or those in which previous endovascular treatment had failed [1]. The exact mechanism of how an aneurysm is excluded is not completely understood; however, it likely involves several factors that occur in conjunction with one another and allow eventual healing of the underlying parent vessel [2]. These devices share common design features such as a braided wire design and increased metal coverage, approximately 30–40% higher than in conventional stents [3, 4].

A variety of different flow diverting stents (FDS) are currently available and these include the Pipeline Embolisation Device (PED) (Medtronic, Dublin, Ireland), Silk (Balt Extrusion, Montmorency, France), Surpass (Stryker Neurovascular, Fremont, CA, USA), p64 (phenox, Bochum, Germany) and the Flow Re-direction Endoluminal Device (FRED, Microvention, Tustin, CA, USA). Newer derivations of these devices, such as the Pipeline Shield, have also entered the market promising low thrombogenicity [5] with other devices, such as the FRED Jnr (Microvention) and Silk Vista Baby (Balt) designed for smaller vessels. In the past, flow diversion was not considered for use in the distal circulation due to the relatively superficial anatomy and good accessibility for neurosurgical clipping. Similarly, the delivery of devices via larger delivery catheters, typically 0.027 inch internal diameter (ID), to the distal circulation was considered technically challenging [6]. The introduction of devices such as the Silk Vista Baby and FRED Jnr. has now made the treatment of distal aneurysms more feasible.

This article presents the single center results of a newly developed flow diverter, the p48MW (p48 movable wire, phenox, Bochum, Germany) that has been designed to treat distal vessels with diameters between 1.75 and 3 mm. The feasibility, safety, and effectiveness of this treatment are presented.

## The p48MW Flow Diverter

**Fig. 1:**
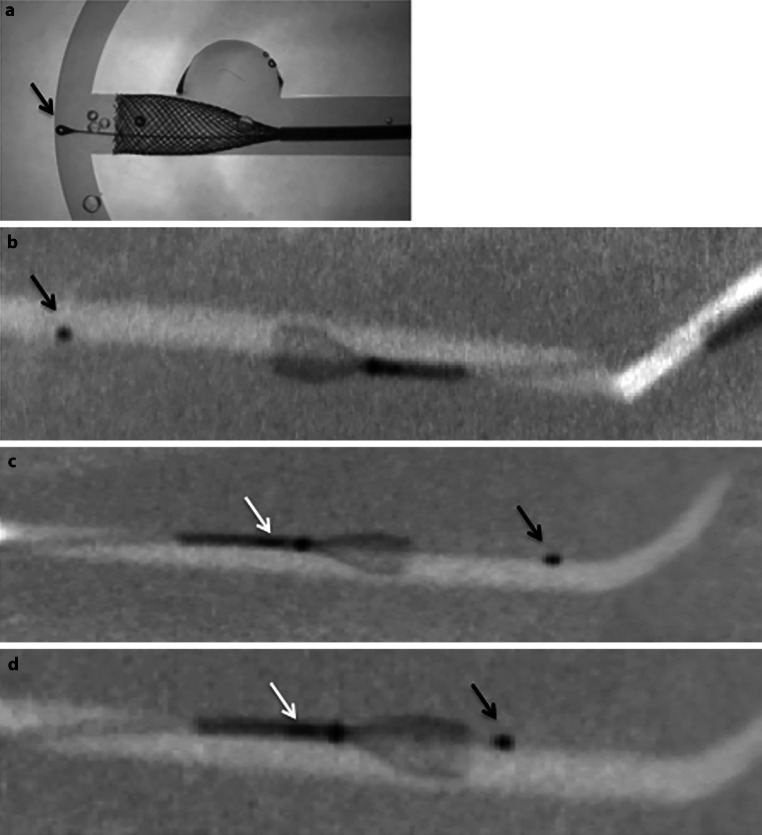
The p48MW has an independently movable central wire with an atraumatic tip (**a**, *black arrow*) that is clearly visualized under fluoroscopy (**b**, *black arrow*). There is a radio-opaque marker that delineates the point of no return after which the stent cannot be recapture (**b**, *white arrow*). One of the key features of the p48MW is the central, independently movable wire. The atraumatic tip can be advanced/withdrawn by the operator during the procedure (**c**, **d**, *black arrow*). The radio-opaque marker (**c**, **d**, *white arrow*) delineates the point of no-return for device deployment

**Fig. 2:**
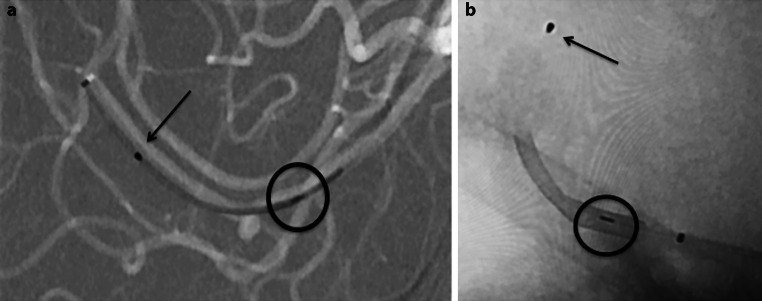
The atraumatic distal tip can be clearly seen within the delivery microcatheter (**a**, *black arrow*) as well as during deployment (**b**, *black arrow*). The proximal marker (**a**, **b**, *black circle*) can also be easily seen within the microcatheter and after deployment allowing for accurate device positioning

The p48MW flow diverter comprises 48 braided drawn filled tubes (DFT) with each DFT strand constructed from platinum-filled nitinol tubing. Unlike the p64 device, the p48 is not mechanically detached; however, a proximal radio-opaque marker identifies the point at which the device can still be re-sheathed. There is a central, independently moveable wire (p48MW) that is compatible with 0.021 inch ID microcatheters and an alternative version without a central movable wire (p48) that is compatible with 0.017 inch ID microcatheters. The movable inner wire is made of nitinol and has an atraumatic distal tip to prevent small distal vessels and perforator branches from rupturing. The p48 is designed to treat vessels of between 1.75 and 3 mm in diameter (Figs. 1 and 2).

## Material and Methods

### Patient Population

A retrospective analysis of the prospectively maintained database was performed to identify all patients who had been treated with the p48MW flow diverter from a single center. The data set included patients with saccular, dissecting, and fusiform aneurysms, both ruptured and unruptured. The procedures took place between January 2017 and January 2019.

For each patient demographic data, clinical presentation, location of the aneurysm, therapeutic intervention, immediate angiographic and clinical results, and clinical and radiological follow-up information were recorded.

### Endovascular Treatment

All treatment was performed with the patient under general anesthesia. Patient informed consent was obtained before the procedure in all cases. All patients with unruptured aneurysms received dual antiplatelet therapy (aspirin 100 mg daily and prasugrel 10 mg) for at least 7 days prior to the treatment. The effectiveness of the antiplatelet regimen was tested using VerifyNow (Werfen, Barcelona, Spain). The post-procedural antiplatelet regimen consisted of clopidogrel/prasugrel continued for 6 months following treatment and aspirin continued for life. For ruptured aneurysms, depending upon the clinical state of the patient, pre-medication was given either on the angio table or orally at least 3 h prior to the operation for patients able to take oral medications. For those patients able to take oral medications, loading doses of aspirin (500 mg) and prasugrel (60 mg) were given on the morning of the surgery. In those unable to take oral medication prior to the operation an intravenous bolus dose of weight-adjusted tirofiban was given on table. Subsequent loading doses of prasugrel via NG tube (60 mg) and IV aspirin (500 mg) were given at the end of the procedure. The effectiveness of the antiplatelet medication was tested 24 h post-procedure using the VerifyNow and Multiplate (Roche, Basel, Switzerland) analysers.

All procedures were performed via the right common femoral route using a tri-axial access system comprising of a 6 Fr Flexor Shuttle long sheath (Cook Medical, Bloomington, IN, USA) and 6 Fr 0.72 inch Navien distal access catheter (Covidien, Mansfield, MA, USA). Either a Trevo 21 (Stryker, Kalamazoo, MI, USA) or Prowler Select Plus (Codman, Raynham, MA, USA) microcatheter was used for access to the parent vessel and deployment of the p48 MW flow diverter. All procedures were performed under heparin anticoagulation with a 5000 IU bolus dose at the start of the procedure and subsequent 1000 IU bolus doses every hour to maintain the activated clotting time between 2–2.5 times the baseline.

### Procedural Assessment and Follow-Up

Patency and flow characteristics within the parent vessel and any covered cortical branches or perforators were assessed angiographically immediately after having placed the FDS and during follow-up. Procedural follow-up, using either catheter digital subtraction angiography (DSA) or magnetic resonance angiography (MRA) was performed initially at 3–6 months (early follow-up), again at 9–12 months (intermediate follow-up) and then once per year. Standard angiographic projections were used to assess the patency of the vessels and the aneurysms in addition to angiographic projections that repeated those used during the treatment. Aneurysm occlusion was graded using the 3‑point Raymond-Roy classification [7]. Neurological examinations were performed to test for potential ischemic or hemorrhagic complications in the postoperative period (<24 h post-procedure) and at each subsequent follow-up.

## Results

### Baseline Demographics and Aneurysm Characteristics

A total of 25 patients (20 female) with average age 55 ± 12.9 years (range 34–84) with 25 aneurysms were identified. The majority of the aneurysms was located in the anterior circulation (19/25, 76%) and on the left (13/25, 52%). The M1 (5/25, 20%) and A1 (4/25, 16%) segments were the most common locations for the aneurysms followed by the P1 (3/25, 12%) segment and PICA (3/25, 12%). Of the aneurysms (32%) were thought to be dissecting in nature, 1 (4%) was classified as a blister aneurysm and the remaining 16 (64%) were saccular. The majority of the aneurysms were incidental and found during the investigation of other conditions, such as headache, stroke, or transient ischemic attacks (TIA). Of the aneurysms 5 (20%) were treated following rupture of another intracranial aneurysm and 4 (16%) were treated acutely after subarachnoid hemorrhage. The average aneurysm dome width was 3.98 ± 3.6 mm (range 1.2–13 mm), dome height was 4.71 ± 5.5 mm (range 1–20 mm), and neck width 2.98 ± 1.4 mm (range 1.5–8 mm). The average aspect ratio was 1.31 ± 0.82 and bottleneck factor 1.22 ± 0.64. The average proximal vessel diameter was 2.5 ± 0.6 mm (range 1.5–3.5 mm) and the average distal vessel diameter was 2.1 ± 0.5 mm (range 1.3–2.8 mm). In all but one case only a single p48MW was deployed. In one case a 2nd device was deployed after ineffective placement of the 1st stent. In a single patient adjunctive coiling of the aneurysm was performed. The results are summarized in Table 1.

**Table 1:** Parent vessel, aneurysm and device characteristics

Patient no.	Aneurysm characteristics											Device characteristics		Adjunctive treatment
	Location	Laterality	Morphology	Proximal parent vessel diameter (mm)	Distal parent vessel diameter (mm)	Neck (mm)	Dome height (mm)	Dome width (mm)	Aspect Ratio	Bottleneck factor	Presentation	No. of devices implanted	Device size	
1	M1	R	Saccular	2.6	2	3.2	4.1	1.7	1.28	0.53	Headache	1	2.5 × 9	No
2	PICA	R	Dissecting	1.4	1.4	2.6	2.6	1.6	1	0.62	Headache	1	2.5 × 9	No
3	P1	L	Dissecting	2.3	2.2	2	2.1	2	1.05	1	SAH other aneurysm	1	3.0 × 15	No
4	A1	L	Dissecting	2.8	2.6	4.6	21	16	4.57	3.48	Stroke	1	3.0 × 12	No
5	MCA Bif	R	Saccular	2.3	1.6	3	3.3	3.2	1.1	1.07	Incidental	1	3.0 × 9	No
6	M1	R	Saccular	2.5	2.3	2.6	4.1	3.2	1.58	1.23	Incidental	1	3.0 × 9	No
7	M1	R	Dissecting	2.7	2.3	2.5	1.5	2.5	0.6	1	SAH	1	2.0 × 9	No
8	PICA	L	Dissecting	4	2.7	8	20	13	2.5	1.63	SAH	1	2.0 × 9	No
9	A1/A2/Acom	R	Saccular	2.6	2	3.6	6.4	6.7	1.78	1.86	SAH	2	2 × 12 (× 2)	2nd p48
10	Acom	M	Saccular	2.2	1.8	3	2	4	0.67	1.33	Incidental	1	2.0 × 9	No
11	A1	L	Saccular	3.2	1.7	1.7	1	1.3	0.59	0.76	TIA	1	3.0 × 12	No
12	P1	L	Dissecting	2.3	1.7	3.5	4	4	1.14	1.14	TIA	1	2.0 × 15	No
13	MCA Bif	R	Saccular	2.6	2.6	2	3.6	2.7	1.8	1.35	Headache	1	3.0 × 15	No
14	A1	L	Saccular	3.5	2.5	2	2.5	2	1.25	1	Headache	1	3.0 × 15	No
15	A1	L	Saccular	3.3	2.8	1.5	2	2	1.33	1.33	Headache	1	2.0 × 7	No
16	M2	L	Saccular	1.9	1.7	1.6	1.3	1.2	0.81	0.75	SAH	1	2.0 × 7	No
17	Pericallosal	L	Saccular	1.5	1.3	4	2.3	3.6	0.58	0.9	SAH other aneurysm	1	2.0 × 15	No
18	Acom	M	Saccular	2.8	2.2	2	3.2	2	1.6	1	SAH other aneurysm	1	3.0 × 18	No
19	Carotid Bif	L	Blister	3.3	2.8	2.8	2	2.7	0.71	0.96	Headache	1	3.0 × 18	No
20	M1	R	Saccular	1.9	2.1	3	4.5	4.5	1.5	1.5	Stroke	1	2.0 × 15	No
21	M1	L	Saccular	3.1	2.8	3.5	3.5	4	1	1.14	Other	1	2.0 × 15	No
22	P1	R	Dissecting	2.1	1.6	3.2	2	2	0.63	0.63	Incidental	1	2.0 × 12	No
23	MCA Bif	R	Saccular	2	1.6	4.5	4	3	0.89	0.67	SAH other aneurysm	1	2.0 × 15	No
24	PICA	L	Dissecting	2.4	2.1	2	3	5	1.5	2.5	SAH other aneurysm	1	2.0 × 7	cCoils
25	Pericallosal	L	Saccular	2.3	1.7	2.5	3	3	1.2	1.2	Stroke	1	2 × 12	No

*R* right, *L* left, *SAH* subarachnoid hemorrhage, *PICA* posterior inferior cerebella artery, *MCA* middle cerebral artery, *bif* bifurcation, *Acom* anterior communicating artery

### Radiographic and Clinical Follow-up

A single patient died before any follow angiographic imaging could be performed. Of the remaining 24 patients, early angiographic follow-up was available for 20 patients (83%) on average at 4.65 ± 1.5 months post-procedure (range 3–6 months). Of the patients 4 had MRA and the remaining 16 patients had DSA at this time point. Aneurysm occlusion was recorded as RRC I (complete occlusion) in 15 cases (75%), RRC II (neck remnant) in 0 (0%) and RRC III (continued aneurysm dome filling) in the remaining 5 aneurysms (25%).

**Fig. 3:**
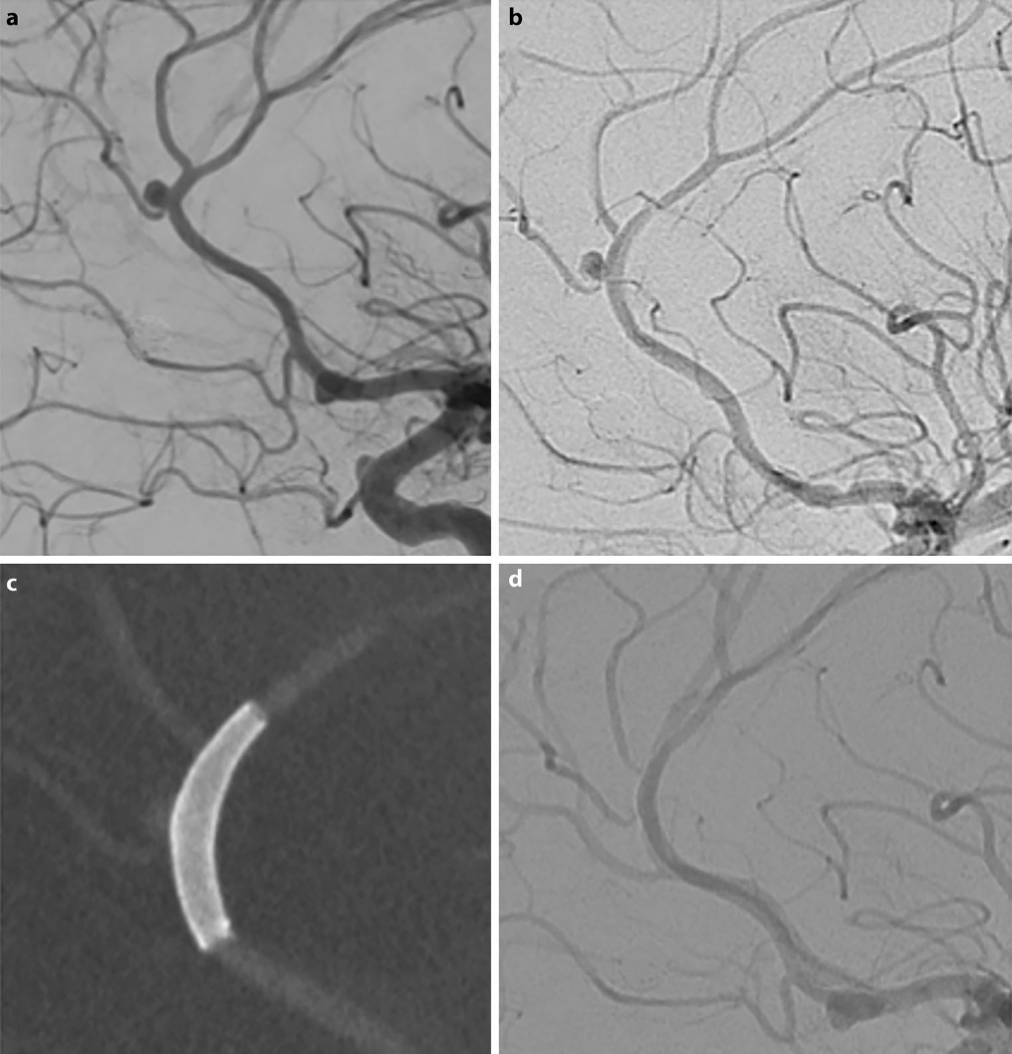
A patient (patient no. 25) with multiple intracranial aneurysms found during investigation for a stroke underwent treatment of a pericallosal aneurysm (**a**) using a single p48MW 2 × 12 mm. There were no intraprocedural complications. Persistent flow was seen within the aneurysm at the end of the procedure (**b**). The device is well seen on flat panel rotational angiography (**c**) thanks to the DFT construction and flow can be seen within the covered branches as well as the treated aneurysm. On follow-up angiography the aneurysm is completely excluded from the circulation (**d**)

**Fig. 4:**
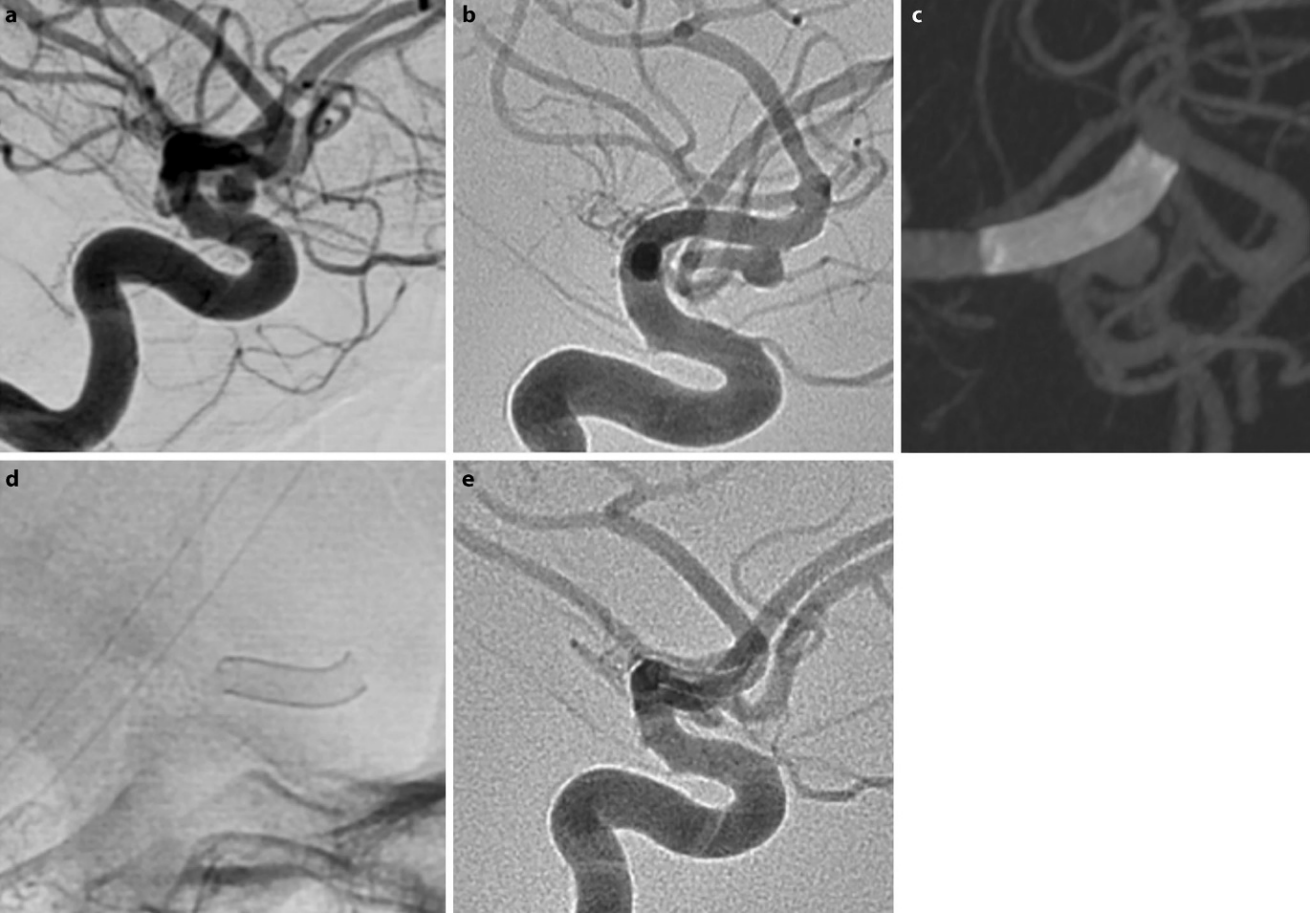
Patient 5 had an unruptured right MCA bifurcation aneurysm that was found incidentally (**a**). After discussion with the patient they opted for endovascular treatment despite the low risk of rupture. A single p48MW 3.0 × 9 mm was deployed with evidence of good wall apposition and continued filling in the covered branch (**b**, **c**). The device is clearly visualized on both flat panel rotational angiography (**c**) and fluoroscopic imaging (**d**). There was persistent filling of the aneurysm dome on the follow-up angiography performed at 12 months; however, this had decreased following treatment (**e**)

At intermediate follow-up, available for 20 patients (83%) at 13.1 months (range 12–24 months), available in 20 patients at an average of RRC I occlusion was seen in 14 patients (70%) (Fig. 3), RRC II in 1 patient (5%), and RRC III in 5 (25%). At any time point complete occlusion was seen in 18/24 (75%) of aneurysms with neck remnants in 1/24 (4.2%) and continued aneurysm filling seen in the remaining cases (5/24, 20.8%) (Fig. 4). Adequate occlusion was therefore seen in 79.2% of aneurysms (RRC grade I or II) during the follow-up period. None of the ruptured aneurysms treated with the device re-ruptured. The results are summarized in Table 2.

**Table 2:** Clinical and radiographic follow-up information

Patient no.	Procedural complications		Angiographic Follow-up					Clinical Follow-up			Comments
	TECHNICAL COMPLICATION	CLINICAL COMPLICATION	Early (avg. 4.65 months)	Intermediate (avg. 13.1 months)	In-stent stenosis	Fate of Covered Branch	Fish Mouthing	mRS Pre-op	mRS Post-op	90 day mRS	
1	No	No	I*	I	No	Patent	No	0	0	0	–
2	No	No	NA	I	No	Patent	No	0	0	0	–
3	No	No	I	I	No	Patent	No	0	0	0	–
4	No	No	I	I	No	Patent	Yes	3	3	3	–
5	No	No	III	III (smaller)	No	Patent	No	0	0	0	–
6	No	No	NA	III	No	Patent	No	0	0	0	–
7	No	No	I	I	No	Patent	No	0	0	0	–
8	No	No	I	I*	No	Patent	No	0	0	0	–
9	Misplacement	No	NA	II	No	Patent	No	1	1	0	1st p48 misplaced so 2nd p48 placed
10	No	No	NA	I	No	Patent	No	1	1	1	–
11	No	No	I	I*	No	Patent	No	1	1	0	–
12	No	No	I	I*	No	Patent	No	1	1	0	–
13	No	No	III	I	No	Patent	No	1	1	0	–
14	No	No	I	I	No	Patent	No	0	0	0	–
15	No	No	I	NA	No	Patent	No	0	0	0	–
16	No	No	NA	NA	NA	NA	NA	5	5	6	Status epilepticus
17	No	No	III	III	No	Patent	No	0	0	0	–
18	No	No	I*	I	No	Patent	No	0	0	0	–
19	No	Yes	I*	I	No	Patent	No	0	0	0	–
20	No	No	III	III	No	Patent	No	1	1	1	Re-treated with p64
21	No	No	III	III	No	Patent	No	0	0	0	–
22	No	No	I	NA	No	Patent	No	0	0	0	–
23	No	No	I	NA	No	Patent	No	1	1	1	–
24	No	No	I*	NA	No	Patent	No	0	0	0	–
25	No	No	I	I	No	Patent	No	0	0	0	–

### Procedural and Clinical Complications

**Fig. 5:**
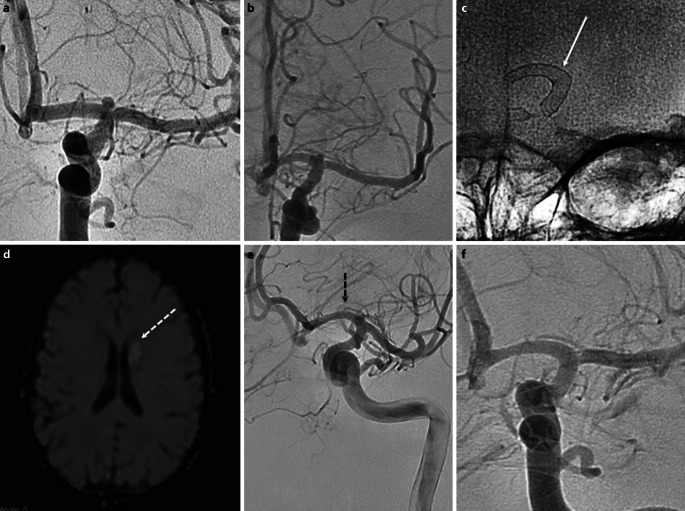
An incidental carotid bifurcation aneurysm (**a**) was found in a patient (patient no. 19) undergoing investigation for headache. After discussion with the patient endovascular treatment for the aneurysm was performed that involved deployment of a single p48MW 3.0 × 18 mm from the A1 and back into the terminal ICA crossing the aneurysm origin (**b**, **c**, *white arrow*). There were no intraoperative complications; however, the patient was found to have unilateral weakness postoperatively. An urgent MRI revealed diffusion restriction within the caudate head (**d**, *dashed white arrow*) suggestive of infarction within the territory of the artery of Heubner. A repeat catheter angiogram was performed which revealed patency of the FDS and the Heubner artery (**e**, *dashed black arrow*). The patient made a complete recovery and the mRS was 0 at 90 days and at last clinical follow-up (12 months). The aneurysm was completely excluded from the circulation at follow-up angiography (12 months) (**f**)

There was only one single procedural complication (4%) and in this case the 1st p48MW stent was not optimally placed, requiring a second device to be deployed. There was a single clinical complication (4%) in a patient treated for a carotid bifurcation aneurysm (Fig. 5). A single p48 device was placed from the A1 into the terminal ICA across the neck of the aneurysm without any procedural complications. Follow-up imaging revealed acute diffusion restriction within the caudate head. In retrospect, the Heubner artery appeared to be slightly covered by the p48MW and it was thought this was the likely cause of the ischemia. The patient made a full recovery and the modified Rankin scale (mRS) score was 0 at 90 days. One patient in our series died (4%). This patient presented with acute SAH and only a small M2 aneurysm was found. The aneurysm was treated after 10 days with no intraoperative or perioperative complications; however, the patient developed uncontrollable seizures while on the intensive care unit that resulted in death on day 15 post-ictus.

### Retreatment

A single aneurysm underwent repeat treatment with a p64 flow diverter (phenox, Bochum, Germany). This aneurysm, arising from the M1 segment of the MCA was treated with a single p48MW. After 9 months there was no significant change in the angiographic appearance of the aneurysm and therefore a repeat treatment with a single telescoped p64 was performed. A repeat angiography of the aneurysm is expected in due course.

### Morphological Changes, Intimal Hyperplasia and In-stent Stenosis

There was no evidence of intimal hyperplasia or in-stent stenosis in any of the cases at follow-up. A single case demonstrated mild fish-mouthing at the distal end of the device but there was no evidence of flow restriction. In a single case (4%) coverage of the Heubner artery was thought to have resulted in a periprocedural stroke; however, the vessel itself remained patent on follow-up angiography. There was no evidence that branch patency was related to continued aneurysm filling.

## Discussion

The use of flow diverters in the intracranial circulation has been widely accepted among the interventional and neurosurgical communities. The Pipeline for Uncoilable or Failed Aneurysms (PUFS) trial, published the initial results in 2013 and showed 73.6% aneurysm occlusion at 6 months [1] with progressive aneurysm occlusion seen reaching 95.2% (60/63) at 5‑year follow-up [8]. The early use of flow diversion was centered on the treatment of aneurysms arising from the internal carotid artery (ICA) with 79/108 (73.1%) aneurysms treated in the PUFS study arising from either the cavernous or ophthalmic segments of the ICA and the majority of the aneurysms being between 10 and 25 mm in size (78.7%). Since the publication of this pivotal trial, numerous reports on the use of flow diversion distal to the circle of Willis have been published [9–25]. Ravindran et al. [26] recently reported their multicohort study of FDS use distal to the circle of Willis defined as at or beyond the A1, M1 and P1 segments of the ACA, MCA and PCA, respectively. They identified 46 patients with 46 aneurysms all treated with either the PED or the FRED from 3 centers. Just under three quarters of the aneurysms (74%) were located in the anterior circulation. The mean follow-up time was 13.0 months at which time complete or near complete occlusion was seen in 36 aneurysms (76.1%). All patients had a good functional outcome (mRS 0–2) and there were 2 cases of perforator vessel stroke (4.3%) but no hemorrhagic complications. Yan et al. [27] sought to determine the efficacy of flow diversion in small caliber vessels. In their meta-analysis, comprising 26 non-comparative studies and 572 aneurysms, the overall technical success rate of the FDS treatment reached 96% (95% confidence interval, CI = 0.93–1.00) with good long-term clinical outcome seen in 96% (95% CI = 0.93–0.99). At last follow-up the complete occlusion rate was 70% (95% CI = 0.64–0.76). Procedure related morbidity was 9% (95% CI = 0.07–0.12) and mortality was 4% (95% CI = 0.00–0.08). Interestingly, there was no statistically significant difference in aneurysms treated with a single FDS or overlapping FDSs although rates of occlusion were higher for the overlapping FDSs (68% vs. 85%, *p* = 0.07) and this would suggest that the design of the stents could be better optimized to target the smaller vessels. It should be noted that the devices used in these studies were likely to have been oversized.

As a result of the braided design of flow diverters, oversizing will result in lengthening of the device [28] which in turn can make accurate positioning, particularly in the proximal landing zone, difficult. Perhaps more importantly, inappropriate sizing of the devices will affect the porosity of the devices. The metal coverage of the devices varies as a parabolic function of the ratio between the vessel and the device and typically oversizing of a FDS will result in reduced metal coverage and increased porosity that could theoretically result in a reduced aneurysm occlusion rate [28, 29]. The inherent oversizing that would have been unavoidable with previous iterations of flow diverter, therefore, may have accounted for the relative low aneurysm occlusion rates seen in the study of Yan et al. [27] as well as the higher rate of occlusion seen when overlapping stents were used. Similarly, inappropriate sizing may alter the patency of any covered side branches which is more likely to occur when these devices are used in the distal circulation [30]. This also suggests that there is a need to developed stents that are specifically targeted to the smaller calibre vessels.

Several devices have recently entered clinical use specifically targeting smaller vessels. These include the FRED Jnr, the Silk Vista Baby and the p48. To date, there are limited data on both the FRED Jnr and the Silk Vista Baby. Möhlenbruch et al. [31] published their multicenter results of 42 patients with 47 aneurysms. The FRED Jnr was successfully deployed in 39/42 (93%) and the vast majority of the aneurysms treated arose from the anterior circulation (42/47, 89.4%) with 18 aneurysms located on the MCA and 24 located on the ACA. The majority of the aneurysms were saccular (*n* = 35) with 9 fusiform aneurysms and 2 giant aneurysms. One aneurysm was treated in the sub-cute period following SAH. Of the aneurysms five represented recurrences following previous coiling, four were regrowths following aneurysm clipping, and two had previously been treated with the PED but had failed to occlude. The median diameter of the aneurysms was 6 mm (range 1.3–25.2 mm) with a median neck diameter of 4 mm (range 1.3–14.5 mm). The median parent vessel diameter was 2.4 mm (range 1.4–3.6 mm) proximally and 2.1 mm (range 1.5–0.3.4 mm) distally. Angiographically, complete occlusion of the 70% of the aneurysms (19/27) was seen at 6 months. Complete occlusion of 73% (8/11) of the aneurysms was seen at 12 months and near complete occlusion was seen in 27% (3/11) of aneurysms. Overall, 78% of aneurysms showed complete or near complete aneurysms occlusion at any time point postoperatively. In terms of safety, the primary endpoint of the study was the absence of mortality, stroke (major or minor) and TIA, with 93% of patients (39/42) achieving the primary endpoint. Side branch occlusion was documented in two patients and in both cases i.v. tirofiban injection was successful in achieving recanalization. Rautio et al. [32] recently published their results with 6‑month follow-up (*n* = 15, 11 female). Of the 15 aneurysms, 8 had previously ruptured and been treated with coiling (*n* = 7) or neurosurgical clipping (*n* = 1) with retreatment required for residual neck or aneurysm regrowth. In all cases the procedure was technically feasible. Adequate wall apposition was seen in all cases and contrast stagnation (O’Kelly Marotta grade B) at the end of the procedure was seen in 2 of the aneurysms (13%). The National Institutes of Health Stroke Scale (NIHSS) and mRS remained stable in all patients during the follow-up period. At 3–6 months follow-up 9 aneurysms (60%) showed complete occlusion and based on the most recent angiographic results for each patient (6–24 months) the number of aneurysms that had been completely occluded reached 13/15 (87%). The remaining two aneurysms had decreased in size. It is of note that the authors reported no branch occlusions or significant caliber reductions in the stented vessels although this remains of concern [18, 33, 34]. Early results on the Silk Vista Baby device have also recently been published [35, 36]. Martinez-Galdámez et al. [35] recently reported the results of a multicenter study involving 41 patients (28 female, average age 50.5 years) with 43 aneurysms. The majority of the aneurysms were saccular (*n* = 30, 69.76%) and <10 mm (*n* = 30, 69.76%). The average artery diameter was 2.28 mm (range 0.9–3.6 mm), the proximal parent artery diameter was 2.8 and 2 mm distally with the majority of the aneurysms located in the anterior circulation (*n* = 34, 79.1%). An average of 1.19 stents was deployed. Multiple devices were used in 6 cases and adjunctive coiling was performed in 13 cases. There were 5 intraprocedural complications, none of which resulted in permanent clinical sequelae. Immediately after stent deployment, 8 aneurysms (18.6%) were classified as completely occluded (O’Kelly Marotta grading system [OKM] D), 5 (11.6%) were near complete occlusion (OKM C), 4 (9.3%) showed incomplete filling (OKM B), and 26 (60.4%) showed complete filling (OKM A). Longer term follow-up data are awaited and in the only other available publication regarding the Silk Vista Baby both aneurysms were completely occluded by follow-up [36]. The results of the present study are very similar with similar rates of occlusion when compared to the FRED Jnr (79.2% p48MW vs. 78% FRED Jnr) and similar rates of complication.

The study is limited by its retrospective nature and relatively small sample size. Similarly, the results may not be readily extrapolated to other centers with less experience of flow diversion. Furthermore, there are limited data on large/giant and partially thrombosed aneurysms and therefore extrapolation to these clinical scenarios is difficult alongside limited experience of the use of the device in the acute setting.

## Conclusion

The p48MW represents a next generation flow diverter designed to target smaller vessels distal to the circle of Willis. The device has similar occlusion rates to other flow diverters and is associated with a low complication rate.

## References

[1] Becske T, Kallmes DF, Saatci I, McDougall CG, Szikora I, Lanzino G (2013). Pipeline for uncoilable or failed aneurysms: results from a multicenter clinical trial. Radiology.

[2] Kadirvel R, Ding Y-H, Dai D, Rezek I, Lewis DA, Kallmes DF (2014). Cellular mechanisms of aneurysm occlusion after treatment with a flow diverter. Radiology.

[3] Augsburger L, Farhat M, Reymond P, Fonck E, Kulcsar Z, Stergiopulos N (2009). Effect of flow diverter porosity on intraaneurysmal blood flow. Klin Neuroradiol.

[4] Lieber BB, Stancampiano AP, Wakhloo AK (1997). Alteration of hemodynamics in aneurysm models by stenting: influence of stent porosity. Ann Biomed Eng.

[5] Hanel RA, Aguilar-Salinas P, Brasiliense LB, Sauvageau E (2017). First US experience with pipeline flex with shield technology using aspirin as antiplatelet monotherapy. Bmj Case Rep.

[6] Brouillard AM, Sun X, Siddiqui AH, Lin N (2016). The use of flow diversion for the treatment of Intracranial aneurysms: expansion of indications. Cureus.

[7] Roy D, Milot G, Raymond J (2001). Endovascular treatment of unruptured aneurysms. Stroke.

[8] Becske T, Brinjikji W, Potts MB, Kallmes DF, Shapiro M, Moran CJ (2017). Long-term clinical and angiographic outcomes following pipeline embolization device treatment of complex internal carotid artery aneurysms: five-year results of the pipeline for uncoilable or failed aneurysms trial. Neurosurgery.

[9] Puri AS, Massari F, Asai T, Marosfoi M, Kan P, Hou SY (2016). Safety, efficacy, and short-term follow-up of the use of pipeline embolization device in small (〈2.5 mm) cerebral vessels for aneurysm treatment: single institution experience. Neuroradiology.

[10] Dabus G, Grossberg JA, Cawley CM, Dion JE, Puri AS, Wakhloo AK (2017). Treatment of complex anterior cerebral artery aneurysms with pipeline flow diversion: mid-term results. J Neurointerventional Surg.

[11] Nossek E, Zumofen DW, Setton A, Potts MB, Raz E, Shapiro M (2017). Treatment of distal anterior cerebral artery aneurysms with the pipeline embolization device. J Clin Neurosci Off J Neurosurg Soc Australas.

[12] Clarençon F, Di Maria F, Gabrieli J, Shotar E, Zeghal C, Nouet A (2017). Flow diverter Stents for the treatment of anterior cerebral artery aneurysms: safety and effectiveness. Clin Neuroradiol.

[13] Bhogal P, Martinez Moreno R, Ganslandt O, Bäzner H, Henkes H, Perez MA (2017). Use of flow diverters in the treatment of unruptured saccular aneurysms of the anterior cerebral artery. J Neurointerventional Surg.

[14] Colby GP, Bender MT, Lin L-M, Beaty N, Huang J, Tamargo RJ (2017). Endovascular flow diversion for treatment of anterior communicating artery region cerebral aneurysms: a single-center cohort of 50 cases. J Neurointerventional Surg.

[15] Briganti F, Delehaye L, Leone G, Sicignano C, Buono G, Marseglia M (2016). Flow diverter device for the treatment of small middle cerebral artery aneurysms. J Neurointerventional Surg.

[16] Pistocchi S, Blanc R, Bartolini B, Piotin M (2012). Flow diverters at and beyond the level of the circle of willis for the treatment of intracranial aneurysms. Stroke J Cereb Circ.

[17] Topcuoglu OM, Akgul E, Daglioglu E, Topcuoglu ED, Peker A, Akmangit I (2016). Flow diversion in middle cerebral artery aneurysms: is it really an all-purpose treatment?. World Neurosurg.

[18] Caroff J, Neki H, Mihalea C, D’Argento F, Khalek AH, Ikka L (2016). Flow-diverter Stents for the treatment of Saccular middle cerebral artery bifurcation aneurysms. Ajnr Am J Neuroradiol.

[19] Iosif C, Mounayer C, Yavuz K, Saleme S, Geyik S, Cekirge HS (2017). Middle cerebral artery bifurcation aneurysms treated by Extrasaccular flow diverters: midterm Angiographic evolution and clinical outcome. Ajnr Am J Neuroradiol.

[20] Zanaty M, Chalouhi N, Tjoumakaris SI, Gonzalez LF, Rosenwasser R, Jabbour P (2014). Flow diversion for complex middle cerebral artery aneurysms. Neuroradiology.

[21] Yavuz K, Geyik S, Saatci I, Cekirge HS (2014). Endovascular treatment of middle cerebral artery aneurysms with flow modification with the use of the pipeline embolization device. Ajnr Am J Neuroradiol.

[22] Cagnazzo F, Mantilla D, Lefevre P-H, Dargazanli C, Gascou G, Costalat V (2017). Treatment of middle cerebral artery aneurysms with flow-diverter Stents: a systematic review and Meta-analysis. Ajnr Am J Neuroradiol.

[23] Bhogal P, Chudyk J, Bleise C, Lylyk I, Henkes H, Lylyk P (2018). The use of flow diverters to treat aneurysms of the posterior inferior cerebellar artery: report of three cases. Interv. Neuroradiol..

[24] Bhogal P, Martinez R, Gansladt O, Bäzner H, Henkes H, Aguilar M (2018). Management of Unruptured Saccular aneurysms of the M1 segment with flow diversion : a single centre experience. Clin Neuroradiol.

[25] Bhogal P, Chudyk J, Bleise C, Lylyk I, Perez N, Henkes H (2018). The use of flow diversion in vessels ≤2.5 mm in diameter—A single-center experience. World Neurosurg.

[26] Ravindran K, Enriquez-Marulanda A, Kan PTM, Renieri L, Limbucci N, Mangiafico S (2018). Use of flow diversion for the treatment of distal circulation aneurysms: a multicohort study. World Neurosurg.

[27] Yan Y, Zhu D, Tang H, Huang Q (2018). Safety and efficacy of flow diverter treatment for aneurysm in small cerebral vessels: a systematic review and meta-analysis. World Neurosurg.

[28] Shapiro M, Raz E, Becske T, Nelson PK (2014). Variable porosity of the pipeline embolization device in straight and curved vessels: a guide for optimal deployment strategy. Ajnr Am J Neuroradiol.

[29] Shapiro M, Raz E, Becske T, Nelson PK (2014). Building multidevice pipeline constructs of favorable metal coverage: a practical guide. Ajnr Am J Neuroradiol.

[30] Miller TR, Kole MJ, Le EJ, Cannarsa G, Jones S, Wessell AP (2018). Pipeline diameter significantly impacts the long-term fate of jailed side branches during treatment of intracranial aneurysms. Ajnr Am J Neuroradiol.

[31] Möhlenbruch MA, Kizilkilic O, Killer-Oberpfalzer M, Baltacioglu F, Islak C, Bendszus M (2017). Multicenter experience with FRED Jr flow re-direction Endoluminal device for Intracranial aneurysms in small arteries. Ajnr Am J Neuroradiol.

[32] Rautio R, Rahi M, Katila A, Rinne J (2018). Single-center experience with six-month follow-up of FRED Jr® flow diverters for intracranial aneurysms in small arteries. Acta. Radiol..

[33] Bhogal P, Ganslandt O, Bäzner H, Henkes H, Pérez MA (2017). The fate of side branches covered by flow diverters-results from 140 patients. World Neurosurg.

[34] Rangel-Castilla L, Munich SA, Jaleel N, Cress MC, Krishna C, Sonig A (2016). Patency of anterior circulation branch vessels after pipeline embolization: longer-term results from 82 aneurysm cases. J Neurosurg.

[35] Martínez-Galdámez M, Biondi A, Kalousek V, Pereira VM, Ianucci G, Gentric J-C (2019). Periprocedural safety and technical outcomes of the new Silk Vista Baby flow diverter for the treatment of intracranial aneurysms: results from a multicenter experience. J Neurointerventional Surg.

[36] Bhogal P, Wong K, Uff C, Wadley J, Makalanda HL (2019). The silk vista baby: initial experience and report of two cases. Interv Neuroradiol.

